# Perspectives and experiences of physical activity among autistic adults in middle adulthood

**DOI:** 10.1177/13623613251360862

**Published:** 2025-08-12

**Authors:** James McLeod, Martin Roderick, Mary Hanley, Deborah M Riby, Patrick Jachyra

**Affiliations:** 1 Department of Psychology, Durham University, UK; 2 Department of Sport and Exercise Sciences, Durham University, UK

**Keywords:** autistic adults, neurodivergent-informed physical activity, neuro-normative assumptions, physical activity, qualitative research, trauma-informed approach

## Abstract

Physical activity (PA) participation among autistic adults is low; however, it is unclear why this is the case. To date, little research has included the perspectives of autistic adults during middle adulthood to understand how to support their PA and to understand the complexity of their daily lives. Seventeen autistic adults aged 36–59 years participated in two online, semi-structured interviews (*N* = 34). Reflexive thematic analysis was used to analyze the data, and the socio-ecological model was applied to interpret the themes. Reflexive notes (*N* = 34) made during the research process also were analyzed to support the rigour of the interpretations. Neuro-normative assumptions about autism and PA, the importance of trust and sensory sensitivities shaped PA participation for these autistic adults. The *new* concept of neuro-normative assumptions highlights how pervasive assumptions were damaging, made it difficult to build trust and decreased the predilection of PA participation. This article delivers original and significant knowledge and calls on the field to co-produce neurodivergent-informed PA practices that are reflective of the lives, needs and abilities of autistic adults. We conclude the article by highlighting how a trauma-informed approach may have potential value in building trust and supporting autistic adults in PA.

**Lay Abstract:**

*What is already known?* Autistic adults experience barriers to participating in physical activity (PA), and their rates of participation are low. This is a problem because PA participation can contribute many health and well-being benefits. There is a need for research to better understand how to support their PA participation. Working directly with autistic adults is of utmost importance as minimal research has directly included their perspectives.

*What this article adds*: Seventeen autistic adults from the United Kingdom participated in two online semi-structured interviews (*N* = 34). Interview data were accompanied by reflexive notes which were a way for the research team to provide preliminary analyses and think about the data during the research process (*N* = 34). Together, interview transcripts and memos were analyzed to generate themes across the interview transcripts. We used the socio-ecological model to examine the different components that impact PA participation. The study highlights how neuro-normative assumptions about PA, trust and sensory sensitivities shaped PA participation for autistic adults. A lack of understanding and knowledge among movement professionals about autism, their needs, interests and abilities and trust significantly impacted participation. Assumptions and limited understandings about autism limited participation, as the forms of PA available often were not suitable to the diverse needs, interests and abilities of autistic adults.

*Implications for practice, research or policy:* The insights highlight the importance of co-producing neurodivergent-informed PA practices that are reflective of the needs, strengths and abilities of autistic adults. Finally, we outline how the use of a trauma-informed approach to PA may be valuable in building trust and supporting PA participation. This is the first study to use this approach for PA with autistic adults.

## Introduction

Physical activity (PA) participation for autistic people can confer many benefits. Among autistic young people, improvements in cardiovascular fitness (Sorensen & Zarrett, 2014), sleep quality (Tse et al., 2019), body composition (Shahrasfenghar et al., 2019), social-emotional health (Alhowikan, 2016) and mental well-being have been observed (DeJesus et al., 2020). For autistic adults, PA participation can contribute to improvements in health-related fitness (Lochbaum & Crews, 2003; Sowa & Meulenbroek, 2012), cognitive function (Shahane et al., 2023) and mental well-being (Hillier et al., 2011). Participating in PA has also been associated with supporting difficult behaviours (Elliott et al., 1994), stress (Hillier et al., 2011) and anxiety (García-Villamisar & Dattilo, 2010) among autistic adults. Furthermore, engaging in PA can contribute to a sense of enjoyment (Blagrave et al., 2021) and can potentially serve as an outlet from daily life (Jachyra et al., 2021). This is important as research suggests that being neurodivergent in a neurotypical world can potentially negatively impact quality of life (Finch et al., 2022).

Despite the documented benefits of PA participation, engagement in PA by autistic adults is low (Benson et al., 2018; Hamm & Yun, 2017). Evidence suggests that only 17.6% of autistic adults currently meet the government’s PA guidelines of at least 150 minutes of moderate-vigorous PA or 75 minutes of vigorous PA (Benson et al., 2018) in Canada. Emerging research from the United States suggests that autistic adults have lower levels of PA participation than their neurotypical peers (Hamm & Yun, 2017; Hillier et al., 2020) and experience many barriers to participation (Healy et al., 2021). Supporting PA participation with autistic adults is crucial as research suggests that PA participation can have protective effects on physical and mental health (aan het Rot et al., 2009; Fortuna et al., 2015; Pinckard et al., 2019). Engaging in PA for autistic people has the potential to contribute social and emotional benefits (Campos et al., 2019) and support opportunities to develop skills used in daily living activities (Wright Stein et al., 2022) and inclusion in society (Gregor et al., 2018). Given the low levels of PA participation with autistic adults, research is needed to better understand their reduced activity levels.

To date, there is a lack of research that has focused on the PA participation of autistic adults as much of the research has been conducted with autistic children and adolescents (Piven & Rabins, 2011). The research on autistic adults has included the perspectives of parents (Nichols et al., 2018; Parsons et al., 2024) and small samples of autistic adults (Parsons et al., 2024). As a result, the perspectives of autistic adults remain predominantly absent as little research has directly included their perspectives (Blagrave et al., 2021; Colombo-Dougovito et al., 2020). There is a need for research to elicit the perspectives of autistic adults directly to better understand their perspectives and experiences of PA and how to support their participation. Further to the lack of research drawing on the perspectives of autistic adults, there is a paucity of research that explores the perspectives and experiences of autistic adults during middle adulthood. Research with autistic adults to date has predominantly included autistic individuals younger than 35 years (Kim et al., 2020), older autistic adults up to 78 years of age (Waldron et al., 2023) and cross-sectional research that included participants across the adulthood spectrum (19–65 years) (see Blagrave et al., 2021; Colombo-Dougovito et al., 2020). The lack of research that explicitly focused on middle adulthood with autistic adults is a significant gap as little is known about the daily lives of autistic adults in relation to PA during this life period. Conducting research with this cohort is important as preliminary research with autistic adults suggests further declines in PA participation as they progress throughout adulthood (Hamm & Yun, 2017; Hillier et al., 2020). Given that middle adulthood is an important period to support and promote PA participation among all adults (Nagata et al., 2022), understanding how to optimally support the PA participation of autistic adults during middle adulthood is needed. Conducting this research is timely and significant as autistic adults are at increased risks of experiencing significant health inequalities across numerous domains compared to the general population, such as high rates of physical (Weir et al., 2020) and mental health conditions (Lai et al., 2019) and their sequalae, increased risks for premature mortality (Lunsky et al., 2022) and lower levels of quality of life (Mason et al., 2018).

In addition to the dearth of research directly including autistic adults, there is a lack of research examining the intersecting influences of PA participation. Research by Colombo-Dougovito et al. (2020) highlights how individual factors influence the selection of PA, and participation is conditional on environmental, sensory and social factors. When individual factors are met, Colombo-Dougovito et al. (2020) argue that this facilitates PA participation. Similarly, Blagrave et al. (2021) explain that PA participation is contingent on sensory factors and the importance of having support to facilitate participation in PA. While this research advances our understanding of PA with autistic adults, this previous work has not captured the daily lives and complexity of PA participation. To this end, it is unclear how biological, psychological and social processes experienced by autistic adults converge to impact PA participation. As a result, there is a need then for a deeper examination of PA with autistic adults, and this is echoed by Colombo-Dougovito et al. (2020) who highlight the need to ‘recognize that multiple levels of influence that act upon our behaviors’ (p. 638). Exploring how biological, psychological and social processes converge together has the potential to generate a more comprehensive understanding of PA participation, which heretofore has been absent.

Given the gaps outlined earlier, the purpose of this study was to (a) examine the perspectives and experiences of PA participation among autistic adults in middle adulthood and (b) examine the biological, psychological and social processes which shape PA participation. This article makes four original and significant contributions to the literature on PA participation with autistic adults. First, the article prioritizes the voices and perspectives of autistic adults in middle adulthood, which has been limited to date. Second, the article provides an in-depth examination into the lives of autistic adults and elucidates a thick description of their PA participation by situating PA behaviours within the broader social and cultural relationships they are embedded in. To illuminate the complexity of PA participation and daily lived contingencies of autistic adults in middle adulthood, the new concept of *neuro-normative assumptions* is introduced to the field of PA and autism research. Third, the article provides further depth, breadth and nuance to advancing our understanding of the dynamic nature of PA by illustrating how biological aspects of autism and PA intersect with and are contoured socially to shape participation. Fourth, the article is the first in PA and autism research to draw on a trauma-informed approach (TIA) and highlights how this approach can be used to inform policies and practices oriented at supporting the PA and everyday lives of autistic adults.

## Methodology and methods

This study utilized an interpretive qualitative design (Schwartz-Shea & Yanow, 2013) to generate in-depth explorations of the lives of autistic adults in middle adulthood and their PA participation. Interpretive qualitative research is a methodology that aims to understand and interpret the meanings that people or groups of people assign to their experiences (Smith & Sparkes, 2016). This methodology is ideally suited when conducting research in areas that have been under-researched or under-examined (Jachyra et al., 2019) and is critical to capture the complexities of PA participation. The study consisted of two semi-structured interviews, and this approach was taken for three reasons. First, semi-structured interviews sought to directly seek the perspectives and experiences from autistic adults (Pellicano & Stears, 2011) given that previous research has often included the perspectives of non-autistic people such as parents and professionals. Second, the use of two interviews established a sense of trust with participants by building rapport over time. Finally, two interviews were conducted to seek a deeper and comprehensive understanding of their lives and their PA participation, and this enabled the interviewer to generate data iteratively in novel directions.

## Theoretical framework

To examine how biological, psychological and social processes experienced by autistic adults converge to impact PA participation, this study drew on the socio-ecological model (SEM) (Bauman et al., 2012). This SEM was selected as it enabled the researchers to examine the complexity of PA participation as the SEM recognizes that PA behaviours are complex and take place in the context relationships between people and their broader social and physical environments (Bauman et al., 2012). The SEM focuses on four components: intrapersonal (demographic, biological, psychological, emotional, cognitive), interpersonal/cultural (social support), environmental (financial aspects, enjoyable scenery) and policy level (laws, policies, regulations and codes; Sallis et al., 2006). The use of the SEM is beneficial to identify the processes that impact PA participation as it recognizes that processes at each of these levels contribute to PA behaviours. The SEM has been used in research examining PA participation with autistic adolescents (Arkesteyn et al., 2025) and widely used in PA research with adults in the general population (Paudel et al., 2021).

## Recruitment and procedure

This study was approved by the institutional research ethics committee. Participants were recruited through social media and charities in the United Kingdom (UK). These recruitment channels were mobilized as reaching autistic adults to participate in research can be difficult due to the history of distrust with institutions and researchers experienced by the autistic community (Woods & Waltz, 2019). Purposive sampling (Campbell et al., 2020) was used, and autistic adults were invited to participate in the study if they (a) had a clinical diagnosis or self-diagnosis of autism spectrum disorder, autistic disorder, Asperger syndrome or pervasive developmental disorder not otherwise specified; (b) were at least 35 years old; (c) lived in the UK; (d) were able to communicate in English (written, oral and/or any other communication modality due to the wide-ranging needs and abilities of autistic people) and (e) were able to provide informed consent. The conceptualization of middle adulthood to commence at 35 years of age was informed by Lachman (2004) who explains that there is significant variability in the expected timing of midlife. Although middle age is typically considered to be between the ages of 40 and 60 years (Lachman, 2004), this life period is a very subjective experience and is not uncommon for variability with approximately a 10-year variability on either end (Lachman, 2001).

To ensure suitability, prospective participants were contacted via e-mail and asked to confirm their diagnostic status, age and where they lived. Interviews were informed by an interview guide and asked in order. Interviews were conducted on Microsoft Teams or Zoom. During both interviews, questions were directed by pre-established probes which were used when necessary to facilitate discussions with participants. This approach enabled the primary author to use a dynamic and flexible line of discussion based on the evolving nature of the conversations, with participants ensuring that the discussions remained contextually relevant based on participant responses. A social story with information about the interviewer (primary author) was sent to participants ahead of the interview to build rapport with participants. Content of the social story such as interests in hobbies and activities of the interviewer often stimulated discussion about participants’ own general interests and engagement in PA.

An introductory meeting was held to confirm suitability to participate in the study. After consent was established, the first interview commenced. To build rapport, participants were asked to describe their interests, activities, hobbies and conceptualisations of PA. Over the course of the first interview, questions such as ‘What does a typical week look like for you?’ and ‘What do you like about PA?’ were used to facilitate discussion. The first interview lasted between 15 and 38 minutes, and field notes were written during and after the first interview to reflect on the data generated, conceptualize potential directions and consider follow-up questions for the subsequent interview. As a neuro-inclusive effort, the second interview was scheduled with participants at a time that was convenient for the participant. The second interview sought to understand their daily lives, the role of PA in their life and what potentially constrained them from being active. Participants also were asked what supports might be beneficial to support PA participation. The second interview commenced by asking participants why they engaged in PA and what PA meant to them. Over the course of the second interview, questions such as ‘What barriers to PA participation do you experience?’ and ‘What can be done to better support your participation’ were used to elicit discussions about what influenced, hindered and/or shaped their PA participation. Interviews were 21–82 minutes in duration. Field notes were written during and after the interview. The field notes included reflections on what was said and detailed the interview setting, the tone of language and the nature of interactions with participants (Smith et al., 2025). Field notes were then captured in reflexive notes to detail initial analytical insights across each participant.

## Participant description

In total, 17 autistic adults (see Table 1 for demographic information) with a wide range of PA engagement were included. Participants generally preferred individual forms of PA rather than group-based PA. All names presented in the article are pseudonyms.

**Table 1.** Participant demographic information.

Pseudonym	Age	Gender	Diagnostic status	Age at autism diagnosis	Co-occurring condition(s)	Diagnosis route	Employment status	DA	MDPA	TOPA
Barbra	48	Female	Clinical diagnosis	44	Arthritis, GAD	Not disclosed	Full-time employment	0	0	Not applicable
Sam	46	Male	Clinical diagnosis	46	ADHD	Public	Full-time employment	4	30	Trail running, open water swimming
Haley	50	Female	Clinical diagnosis	44	Arthritis, dyspraxia	Public	Full-time employment	7	60–180	Walking
Georgia	52	Female	Clinical diagnosis	48	Dyspraxia, dyslexia Self-diagnosed with: ADHD	Private	Full-time student	0	0	Not applicable
Jude	48	Female	Clinical diagnosis	48	Dyspraxia, GAD Self-diagnosed with: ADHD	Public	Full-time employment	4	20	Pilates (at home), yoga (at home)
Sarah	46	Female	Clinical diagnosis	45	GAD, Panic disorder, MDD Disordered eating	Private	Full-time employment	5	180	Walking, yoga
Harry	50	Male	Clinical diagnosis	40	Self-diagnosed with: ADHD, hypertension, GAD, acid reflux	Public	Full-time employment	5	30–60	Walking, bodyweight resistance training (at home)
Ellen	53	Female	Self-diagnosis	35	Ehlers-Danlos Syndrome	Not disclosed	Unemployed	0	0	Not applicable
Mary	45	Non-binary	Clinical diagnosis	41	Self-diagnosed with: dyspraxia, hypermobility	Private	Self-employed	3–4	60	Aerobics, yoga
Anna	36	Female	Clinical diagnosis	8	Dyslexia, dyspraxia, sensory processing disorder. obsessive-compulsive disorder	Public	Full-time employment	7	20–40	Walking
Cassie	47	Female	Clinical diagnosis	43	Self-diagnosed with: ADHD, dyslexia, Ehlers Danlos syndrome, disordered eating, chronic migraines	Public	Unemployed	0	0	Not applicable
Brandon	52	Male	Clinical diagnosis	48	Self-diagnosed with: pathological demand avoidance, GAD, MDD, hypermobility	Public	Full-time employment	7	120	Walking
Colin	59	Male	Clinical diagnosis	54	ADHD Self-diagnosed with: obsessive-compulsive disorder, bipolar disorder	Public	Unemployed	7	120	Walking, cycling
Kathleen	49	Female	Clinical diagnosis	46	ADHD, endometriosis, MDD, GAD	Public	Part-time employment	7	45	Walking, cycling
Lindsey	53	Female	Clinical diagnosis	52	Fibromyalgia, MDD, GAD, anorexia nervosa, PTSD, arthritis	Public	Part-time employment	7	30–60	Walking
Valerie	51	Female	Self-diagnosis	Not applicable	Hypermobility Dysautonomia	Not applicable	Unemployed	0	0	Not applicable
Stacey	54	Female	Self-diagnosis	Not applicable	Hypermobility	Not applicable	Self-employed part time	7	60	Running, badminton Household chores, manual labour

ADHD = attention deficit hyperactivity disorder; DA = days active; GAD: generalized anxiety disorder; MDD: major depressive disorder; MDPA = minutes of daily physical activity; N/A: not applicable; PTSD: post-traumatic stress disorder; TOPA = type(s) of physical activity.

## Data analysis

All interviews were recorded with Otter (2023) transcription and manually checked for accuracy of transcription. Analysis of the interviews (*n* = 34), field notes from the interviews and reflexive notes (*n* = 34) which included summaries of analysis for each participant was guided by reflexive thematic analysis (Braun & Clarke, 2019, 2020). The analysis commenced by reading and re-reading all interview transcripts (Braun & Clarke, 2019), field notes and reflexive notes. Interview transcripts were manually annotated with inductive codes. Inductive codes were then cross-examined across participants to look for patterns of meaning across segments of the data conducted by the first and last author. Next, codes were grouped into categories and discussed across a series of meetings with the research team to examine similarities, patterns and exceptions (Braun & Clarke, 2019). Analysis of exceptions (data that deviated from the emerging plotline of the analysis) was critical to enhance the *rigour* of the interpretations. Analyzing exceptions provided new ways of seeing and analyzing the data and to take the data into fertile and unexpected directions (Smith & Sparkes, 2016). Through analytical discussions with team members, categories were refined further until consensus was reached and candidate primary themes were established and named.

Next, the SEM (Bauman et al., 2012) was applied to the themes to further interpret the qualitative data. Using a classification matrix for each component of the SEM, each theme and supporting evidence of each theme was categorized to the corresponding SEM component(s) and into their respective levels of the SEM. Once all themes and supporting evidence were classified into the four components of the SEM, we examined the interconnections by querying how themes and analysis categories converged, diverged or diffracted from each component of the SEM. Interconnections between biological, psychological and social influences of PA were noted with multiple SEM categories as they pertained to the specific behaviour(s). Analysis was complete when the SEM was applied to all themes generated during the first part of the analysis. The diverse expertise of the research team strengthened the rigour of the analysis, with training in sport and exercise sciences, qualitative research, sociology, psychology, psychiatry and critical social science research.

## Results

For autistic adults, engaging in PA was shaped by three interconnected themes: *neuro-normative assumptions about physical activity and autism, trust and sensory sensitivities*. While the three themes are presented independently to help with readability of the paper, the processes are interdependent and highlighted in detail below.

## Neuro-normative assumptions about PA and autism

Across the accounts, neuro-normative assumptions drew on predominant bio-medical conceptualizations of autism and PA. Neuro-normative assumptions also included a strong focus on using PA to alter one’s bodily physique and combined with bio-medical conceptualizations of autism, contributed to a lack of understanding, knowledge and individualization of PA among movement professionals. Neuro-normative assumptions however diverged from the reasons as to why these particular autistic adults engaged in PA. Across the accounts, participants shared how the notions of health and exercise from movement professionals such as personal trainers (PTs) often conflicted with their reasons to participate in PA. For participants in this study, engagement in PA often drew on dominant societal logics of practice centred on altering bodily aesthetics or appearance or maximizing physical performance (environmental processes). Pushing the physical limits of the body, competition and winning also were common norms, values and assumptions shared by movement professionals as to why one should engage in PA. Neuro-normative logics were highlighted in the narratives of Haley and Anna who expressed:

Gyms and the classes are very much focused on you can see that the outcome is very much centred on losing weight. You know, getting a bikini body, not going to ever have a bikini body. I’m 50 years old. I’m not going to wear a bikini. And I want to be fit but it very much seems focused on somebody else’s idea of like the perfect body.

But I think I’m more annoyed with the teacher, you know, that they wouldn’t kind of think about the obsession with winning and maybe make it less about winning, and how it makes people feel. You know, they need to manage that a bit better.

While this logic of practice was conducive for some participants to support their PA participation, for others, these logics of practice created an apathy towards PA. This was particularly the case when neuro-normative assumptions about PA described earlier did not align with the pursuit of wellness, movement, enjoyment of moving and supporting mental health sought by some participants. For some participants, this deterred them from PA as a whole. Highlighting the impacts of these dominant practices on participation, Sarah and Haley noted:

I try to engage in activity for my well-being and health. There is such a focus by PT’s on losing fat, I guess it’s a capitalist society outward appearance type stuff. They should ask about my goals and not assume I want to change my appearance.

Unfortunately, a lot of trainers that I’ve spoken to are tribal, we’re gonna get you on this programme, and you’re gonna do this and you’re gonna lose this much weight in this much time and I’m like, whoa, I never said I wanted to lose that much weight. You didn’t ask me what I wanted. So I do find that sometimes it’s too weight-loss goal oriented. What I did like when I met my personal trainer, was that she asked me, What’s your goal? This allowed me to focus on my mental health and actually enjoying moving not doing it because I have to lose weight.

Problematically, neuro-normative assumptions had manifold impacts as some participants developed a disdain towards PA and contributed to a reluctance to develop interpersonal relationships with movement professionals. Hayley highlighted that her scepticism towards PTs derived from their predominant focus on ‘losing weight’. For Hayley, Anna, Barbra, Kathleen, Lindsey and Sarah, their participation in PA was curtailed and resulted in complete avoidance of certain forms of PA as these neuro-normative logics of practice were not conducive nor reflective of their daily lived contingencies, needs and abilities. Highlighting the emphasis on winning and competition imposed by movement professionals during PA, Barbra and Lindsey noted:

There was another instructor who was totally obsessed with how I would be beating my personal best . . . he was running alongside me but he was just push, push, pushing . . . (he kept saying) you’re gonna get your PB, you’re gonna get your PB, and I wasn’t bothered about my PB. I’m not one of those people who can beat a PB, if I just got a similar time, I’m happy.

I think it was more that I just learned to avoid or just avoided anything where I thought the instructor were going to be super pumped about their sport. The instructor that I have now isn’t like one of these pumpy pumpy people with the microphone to their chin, you know because that that’s quite intimidating and also quite irritating for autistic people. But so common in the gym.

Neuro-normative assumptions created a PA paradox as participants highlighted how movement could help alleviate some of the co-occurring physical and mental health conditions they experienced. Yet, despite the need to engage with movement professionals due to their co-occurring needs, they were not often able to engage in forms of PA as participants expressed that they felt that movement professionals were unable to adapt their approach to account for their needs (e.g. sensory processing differences, dyspraxia and/or arthritis), verbal processing speed and interests (intrapersonal process). Not only did this contribute to an overall lack of interest in PA, but it also contributed to repeated frustration, as difficulties adapting instructional methods and activities (interpersonal process) drew on dominant neuro-normative conceptualizations of PA which were not conducive to their needs. This PA paradox was illustrated by Anna and Sam who highlighted how the lack of knowledge and understanding among movement professionals to deliver PA to autistic clients, understand their needs and lives, dissuaded participation. They remarked:

My biggest kind of issue was I don’t think that the person teaching the classes made them very individualised, and I don’t think he ever had an awareness of things like dyspraxia . . . I felt like I shouldn’t be teaching you (the personal trainer) how to show exercise to people with additional needs. That’s your job.

Give instructions verbally and in writing as well. If you’re still there saying right, you need to do this, not that, then that is not working for me and how I process things. The written bit is super helpful but lacking and makes it harder for me and probably other people like me to engage in activity overall.

For participants in this study, the systemic lack of understanding among movement professionals about autism, delivering PA to autistic people and those who have co-occurring conditions and needs, was commonplace. This served as a significant interpersonal and policy-level barrier to engage in PA as participants highlighted that movement professionals did not account for the impacts of their co-occurring health conditions and potential needs and abilities. This was a policy-based issue as the practices employed by movement professionals often drew on bio-medical, normative and deficit-based approaches which made assumptions what autistic adults could not do, rather than what they can do. Sarah and Anna reflected in their narratives:

People in the industry don’t get us I think, they don’t get all the things going on with us, health conditions and the many one’s we might have and or even how these conditions make it harder for us to be active. Many of these might be invisible and if they don’t see them they assume we can’t do certain things and if we tell them, then they still assume we’re not good enough or they tip toe around us. A few simple things they can do like asking what our sensory needs are, and what I want to get out of the session as well based on what I can do that particular day. Because for me it would depend on my mood be and how I feel in terms of energy that day and what level of overwhelmed I am in on that specific day. Those affect what kind of session I can do. When you walk into fitness places, it can be quite daunting, and just to get people in physical activity to realise yeah that there’s many reasons why someone might want to do an activity and find ways to just make them comfortable. Those strategies adapted to a specific person go a long way but often are missing.

I kind of got a sense that he was getting a bit frustrated because I was really struggling to follow his verbal instructions, you know, not kind of understanding how to translate what he said to actual movements. Then it was full shut down mode from them, they just stopped engaging with me and assumed I couldn’t do much ever.

Neuro-normative assumptions not only contributed to poor PA experiences but also curtailed future participation as participants feared exacerbating co-occurring conditions (such as Ehlers-Danlos syndrome, proprioception issues) and/or the development of potential injuries. The accounts highlighted how participants were sceptical of movement professionals and experienced difficulties trusting them with their bodies, lives and engagement in PA. The constellation of these intrapersonal, interpersonal and policy processes (due to a lack of policies and systems in place to support the PA of autistic adults) impacted PA participation as a whole. Jude and Valerie noted:

All of the pilates and yoga people are very encouraging, and they don’t want you to hurt yourself. We really want you to take your time and do it really gently. Whereas a lot of other instructors haven’t got that stance at all. This worries me that I will get hurt or make current EDS issues worse and it is safer not to do activity.

I think there’s a huge lack of education, understanding and appreciation of what it is like to support someone who is autistic in this space. . . . This is a problem because you don’t want to go to the expert to be the expert, but this is how it is at the minute. You know you will probably come away worse off and get injured so it might be better to just avoid it.

When asked about ways to improve the PA participation for autistic adults, study participants highlighted the need for movement professionals to transcend body-centred training practices and advocated for person-centred approaches that prioritized client needs and interests. Increasing awareness and knowledge autism, common co-occurring health conditions and potential ways to implement PA for autistic adults was identified as an urgent priority. Notably, participants called for action to combat the entrenchment of the bio-medical paradigm of autism in sport and PA practices, spaces and places. As a potential way to enhance the PA participation of autistic people, participants highlighted the need to adopt new approaches that did not draw on neuro-normative assumptions as previously outlined and ensure that approaches to PA were reflective of autistic adult’s lives, needs and abilities. When asked whether having a movement professional who was trained to work with autistic adults would increase PA participation, Harry and Sarah noted:

So if the personal trainer had that training from a regressive old fashion(ed) probably delivered by a neurotypical in a medical model, then no (it would not).

And a lot the classes themselves having instructors trying to motivate people by shouting at us, but I’ve just found them a bit is a bit too much. Almost shouting at you it’s just too intense. I can’t see how that helps autistic people to be active. It might push them away if anything. We need better ways, this just doesn’t work for us.

As illustrated earlier, neuro-normative assumptions made about PA, autism and co-occurring conditions experienced by autistic adults were contributing interconnected intrapersonal, environmental, interpersonal and policy-level processes that shaped and curtailed PA participation. The identified processes intersected with the importance of feeling safe and trusting movement professionals to do no further physical and or psycho-emotional harm. It is to this discussion that we now turn.

## Trust

Trust was an active, dialectical process identified across participants which drew on previous (positive and negative) experiences with PA and shaped the predilection of future participation. In recollecting their lives, some participants reported traumatic life events in childhood such as bullying in school-based settings such as physical education (PE) classes. One participant experienced sexual violence, and one participant recounted experiences of being sexually objectified in PE classes. These formative experiences had lifelong, interpersonal implications as some participants did not feel safe or at ease interacting with PA environments. Spaces associated with PA were inherently triggering and contributed to the distrust of others, especially when participating in group-based PA. Despite the motivation, intention and capability to be active, it was the combination of interpersonal, intrapersonal and environmental factors that shaped the PA participation of autistic adults. Highlighting the impacts of bullying in PE classes, Georgia highlighted:

I think . . . the effect of bullying and confidence with sport with strangers . . . I think that is a big thing of it is the fact other people are watching you. I don’t know them. When I say don’t know them I mean I don’t know them really well. I don’t trust them or they haven’t got my trust and that makes it really hard. I think that’s just scars.

For Sarah and Mary, their distrust and apathy towards movement professionals (such as PTs) derived from the effects of bullying in PE classes. For example, Mary’s account reveals that her mistrust of PT as a whole was influenced by her previous negative experiences such as that she was ‘fat shamed by the PE teacher’. The impacts of these traumatic life events were illustrated by Jude, who highlighted how the effects of childhood bullying impacted her life:

Because I was bullied in PE there’s no way I was ever going near a trampoline until I was with the bairn until he was little . . . because bullying affected my self-esteem I can’t do hairdressers and I think it’s mostly because of the mirror.

Similarly, Sarah revealed that her distrust towards other PTs who adhere to desirable societal stereotypical notions of health derived from ‘the whole PE teachers fit girl’s thing in PE . . . women are intimidated by someone who looks like one of their school bullies’. Although Sarah was a PT and was highly physically active, the fear of being re-traumatized by the effects of childhood bullying highlights the role of the intrapersonal and interpersonal dimensions of PA. The insights described earlier not only demonstrate the effects of childhood traumas but also highlight the feelings of vulnerability that can be associated with being active. Difficulty trusting movement professionals arose from a lifetime of negative experiences with healthcare and movement professionals where they felt disbelieved, misunderstood or misdiagnosed. This was a novel finding as the repeated negative interactions described by participants contributed to psycho-emotional disablism where participants felt ‘unworthy’ and did not feel valued after repeatedly being told they ‘Are too complex and have complex needs’ (Cassie). Deriding descriptions of their multiple and intersecting needs used by movement professionals were not value neutral, nor were they value free. Rather, dominant narratives used by movement professionals were internalized by some participants who attributed blame to themselves and saw themselves as being the problem and the reason why they had minimal engagement with PA. These bad outcomes of potentially good intentions, further eroded trust with movement professionals and detracted their engagement with PA altogether. The multiple disabling effects of psycho-emotional disablism are evident in Cassie’s account:

For me, it was more of an internal, why can’t you do this? What’s wrong with you? Why can’t you do it? Everyone else is doing this. You put in as much effort, more effort, than everyone else. Why can’t you do it? And I didn’t know that I had Ehlers-Danlos at the time, so it’s more of an internal beating myself up. It annoyed me because people were running alongside going you can do it.

Feelings of vulnerability arose from engaging in spaces and places connected to previous trauma and feelings being ‘watched by others’ when engaging in PA. Furthermore, the athletic type of clothing typically worn in PA spaces also made some participants feel vulnerable, and the tight-fitted nature of the clothing often caused a sensory disturbance and difficulty engaging in PA. Given the vulnerability of PA described by participants, trust was a critical component to be active where participants highlighted the importance of feeling safe. Trust was built incrementally through repeated positive interactions and experiences with people, places and things associated with PA. This was an intrapersonal and interpersonal iterative process where participants ‘took one step forward and two back’ (Cassie) to (re)establish new relationships with PA, people, places and things. For participants in this study, tinkering with (re)forming these relationships was vital to build trust and to potentially participate in PA. Yet, it was difficult to do so as PA sessions often were full of changes with different movement professionals in these spaces, the use of different rooms and different people. Given these constant changes and high degrees of uncertainty often connected with PA, building trust was a long and often difficult process after a lifetime of being on the margins for their neurodivergence and challenging experiences with PA. However, PA environments often were not amenable to the time and particular adaptions needed to build trust, to be physically active. This was particularly the case with sensory sensitivities, which was a critical component of PA participation, as highlighted below.

## Sensory sensitivities

Sensory sensitivities entailed biological, psychological and social elements that interweaved to shape PA participation. Auditory, visual, tactile, olfactory and the interplay of these sensory sensitivities were critical in shaping PA participation. In what follows, the experiences and impacts of these sensory sensitivities are described.

### Auditory sensory sensitivities

Many participants highlighted the significance of auditory sensory sensitivities in shaping their PA experiences in community-based settings such as gyms and leisure facilities. This intrapersonal dimension hindered their PA experiences as sensory sensitivities were associated with feelings of trepidation, aversion and apathy. The intrapersonal dimensions of auditory sensory sensitivities contributed greatly to participants’ experiences of burnout, outside and within the sport and PA context. Many participants shared that these auditory challenges derived from a disconnect between their intrapersonal sensory needs and the dynamics of environmental factors often outside of their control. For example, Harry emphasized how his reluctance to participate in community-based PA stemmed from trepidation towards being unable to ‘filter out’ the sounds. Similarly, Kathleen reflected that her aversion towards participating in community-based PA was associated with her inability to ‘control the noise’. To this end, not only was there a lack of opportunities to be active in spaces and places conducive to their needs, but these negative experiences also shaped their reluctance to participate in community-based PA given the arduous nature of community-based spaces.

Across the accounts, participants also highlighted feelings of overwhelm due to sensory sensitivities. For these participants, the entrenchment of auditory stimuli within community-based PA environments made their PA experiences arduous as they often experienced physical and mental anguish. For Jude and Haley, the intrapersonal challenges associated with community-based PA were exacerbated by multiple auditory stimuli. For instance, Jude described feeling overwhelmed when there were multiple auditory stimuli present ‘I can cope with the music and I can cope with the MTV unless you’ve got two different things on at the same time’. Similarly, Haley described feeling overwhelmed by ‘the 50 different noises’ that are in community-based spaces. The presence of multiple auditory stimuli shaped her reluctance towards participating in community-based PA given that the stimuli often change. For these participants, the stimuli not only were overwhelming, but the environmental context was exacerbated as they had no control in minimizing the stimuli and/or their debilitating effects. As a result, some participants completely avoided community-based PA, and PA altogether, due to overstimulation and/or hypersensitivity. Highlighting the impact of auditory sensory sensitivities, Lindsey noted:

One of the worst things for me is the clanging sounds . . . the clanging sounds of the metal is also very uncomfortable so yeah so my later experience of the gym meant that I let those things stop me going.

Despite recognizing the benefits of participation, participants noted that avoiding PA was more beneficial than burning out altogether. This was especially the case when visual sensory sensitivities also were involved and impacted PA participation as highlighted below.

### Visual sensory sensitivities

Visual sensory sensitivities were another important intrapersonal factor shared by participants. The amount and presence of fluorescent and unnatural lighting in community-based PA environments made participation difficult as it was distracting and distressing for some participants. For Ellen, the need to be able to see more natural ‘light and trees’ was important, as she likened her experiences of visual sensory sensitivities to a ‘panic room environment’. In a similar vein, Haley and Sarah described the need to minimize the amount of fluorescent and unnatural lighting in community-based PA environments and further emphasized that their inclination towards participating in community-based PA would be encouraged by the presence of more ‘natural lighting’ and minimizing the number of ‘white lights’. For these participants, they did not lack the knowledge or motivation to participate in PA. Rather intrapersonal processes (visual sensitivities) were not congruent with broader environmental processes (lighting) which did not take the needs of autistic people into account. This in turn prohibited their engagement in PA and connected to tactile and olfactory sensory sensitivities to which we now turn.

### Tactile, olfactory and the interplay of sensory sensitivities

Some participants described discomfort either from transitioning ‘between wet and dry’ (Anna) or from the material of ‘the carpets’ (Sam) that are typically located in community-based PA settings. For participants such as Sam, not being able to ‘face’ tactile sensory sensitivities curtailed participation in PA. The impact of this was similarly observed across olfactory sensitivities where participants such as Jude highlighted how the ‘rubbery smell’ of trainers and treadmills were repulsive and prohibited her participation. Similarly, Lindsey explained that his olfactory sensory sensitivities to synthetic fragrances such as ‘perfumes, deodorants, (and) soap powders’ prohibited him from accessing and participating in community PA. For participants in this study, not only were there limited opportunities for PA participation, but they were also often unable to participate due to sensory sensitivities. These interconnected intrapersonal and environmental factors not only shaped participation but had manifold somatic impacts. For participants like Valerie, the interplay of multiple sensory sensitivities was mentally demanding where it is like ‘a really complex math problem and it’s using all your mental energy it’s like trying to do ten of those at once’. The mental and physical exhaustion associated with these multiple and integrated sensory sensitivities was significant and prohibited participation in PA altogether as they were already physically and mentally exhausted just from being in that environment. This exhaustion however was often interpreted by others (coaches, parents, family members) as being unmotivated and/or not trying hard enough, which further detracted from engaging in PA. The misunderstanding about the role of sensory features not only detracted from the ability to engage in PA but also damaged trust with PA itself.

## Discussion

This qualitative study examined the perspectives and experiences of PA with autistic adults in middle adulthood. Findings of this study provide four novel, significant and original contributions to the literature. First, this study prioritized the perspectives and voices of this cohort, which has been limited to date. Second, this article provides a deeper and more nuanced understanding of PA and the daily lives of autistic adults to illuminate that participation was not a behaviour solely influenced by processes acting in isolation or lacking motivation to be physically active as reported in previous research with autistic young people (Duquette et al., 2016). Rather, PA participation was shaped by several interconnected processes between the intrapersonal (sensory sensitivities), interpersonal (trust), environmental (sensory sensitivities) and policy-level (neuro-normative assumptions) of the SEM, which is a third contribution of the study. This interconnected understanding highlights the need to ensure PA policies, practices, interventions and supports accounts for these multi-level processes in tandem with individual lived experiences when supporting the PA of autistic adults. The use of a TIA to potentially support PA with autistic adults is described below and is a fourth contribution of this article.

In this study, neuro-normative assumptions about PA and autism shaped PA experiences and participation in myriad ways. Neuro-normative assumptions entrenched in a bio-medical paradigm had multiple effects as autistic adults highlighted that movement professionals had difficulty adapting their instructional methods to accommodate to specific needs, interests and abilities. Neuro-normative assumptions were disabling in and of themselves as autistic adults internalized disabling narratives about their multiple and co-occurring needs. This form of psycho-emotional disablism (Reeve, 2012) is a type of social oppression that impacts a person’s emotional well-being, sense of self, self-esteem and self-confidence. Given these repeated and cumulative negative impacts, an aversion to trust, reticence towards PA and ultimately deterred PA participation were notable across the accounts. Recognizing the manifold processes involved in PA participation for autistic adults, findings of this study highlight how PA participation is more than merely a behavioural phenomenon driven by motivation, conscious choice or having the knowledge about how to be active. Rather, social norms, values and paradigms that inform logics of PA practice, intersect with intrapersonal and interpersonal processes and generate conditions of possibility to engage in PA. This relational understanding of PA participation is critical to highlight the depth and nuance of the lives of autistic people, which heretofore has been missing in the literature. This relational understanding also calls on the academic, policy and practitioner fields to interrogate predominant neuro-normative assumptions about PA and autism. As highlighted in the results, there is a need for a paradigm shift to co-produce *neurodivergent-informed physical activity* (see Jachyra et al., 2025) practices that are neuro-affirming, recognize the strengths of being autistic as well as challenges experienced by autistic people in the world and recognize the presence and impact of multiple co-occurring conditions which may impact PA participation. With no education and training offered to movement professionals on how to best support the PA of autistic adults in the UK (De Lyon et al., 2016), the development of neurodivergent-informed PA practices is timely and significant to support the PA of autistic people across the lifespan. Future work is needed in this area.

This study also illuminated the role of trust in shaping PA participation of autistic adults. This is the first article to identify trust as a component in shaping PA experiences across the life course for autistic adults. This finding is of particular importance as research suggests that autistic people experienced high levels of trauma throughout the life course (Peterson et al., 2019). In childhood, autistic children are more likely to experience high levels of bullying (Cappadocia et al., 2011), victimization among peers (Gibbs & Pellicano, 2023) and adverse childhood experiences (Rigles, 2021). In adulthood, they are at risk of being victimized through interpersonal violence from a family member or friend (Pearson et al., 2022), experiencing othering (Pearson et al., 2022) and experiencing structural inequalities and trauma (Jachyra et al., 2024). As highlighted in the results, victimization throughout their lives had profound impacts on trusting others such as movement professionals. The combination of these life experiences with neuro-normative assumptions of PA negatively impacted their PA participation. Recognizing the importance of trust and the high rates of trauma experienced by this group, the use of a TIA may be beneficial when supporting the PA of autistic adults. A TIA recognizes that trauma is prevalent and has widespread impacts (SAMHSA, 2014). To support people who have experienced trauma and to resist re-traumatization, a TIA draws on six principles to guide policy and practice, which include safety; trustworthiness and transparency; peer support; collaboration and mutuality; empowerment voice and choice; cultural, historical and gender issues. Each of these principles should be considered when conceptualizing, designing and implementing PA for autistic adults to prioritize the psychological and emotional well-being of autistic adults. For example, when utilizing the safety principle of TIA, emotional, psychological and physical safety should be prioritized by considering the tone, delivery of information and form of communication used. Another safety consideration is proximity to the client by thinking through how to best share the same space as in an open and non-threatening way. In unison with the paradigm shift challenging neuro-normative assumptions identified earlier, the use of TIA individualized to each person may be of value to build trust and potentially support PA participation for autistic adults. This person-centred and needs-based focus on PA dovetails with the neurodivergent informed PA approach described earlier and, in an effort, to try and support the PA participation of autistic adults.

Another contribution of this article is adding depth and breadth in understanding the role of sensory sensitivities and their impacts on PA. The interplay of sensory sensitivities and in-depth descriptions and impacts adds further nuance to the role of sensory aspects of PA. Consistent with previous research (Blagrave et al., 2021; Nichols et al., 2018), sensory sensitivities were a common and important component shaping PA participation. While sensory sensitivities have consistently been identified, they have not been described in significant detail. As illuminated for the first time in this study, the role and impact of sensory sensitivities on PA participation were misunderstood by individuals working in PA contexts, and assumptions were made about their PA engagement. As a result, there is a need to further develop the discourse about the role of sensory sensitivities impacting the daily lives and PA of autistic adults. Whereas there has been some research examining how to adapt and change elements of the built environment within various public spaces to account for the sensory sensitivities of autistic adults (MacLennan et al., 2023), little research has captured how best to create sensory inclusive environments in sport and PA contexts to support autistic adults’ PA participation. Given this paucity of research in PA to date, future research exploring how to develop built environments more conducive to the sensory needs of autistic people is of upmost importance to work towards an inclusive society for autistic people.

## Study limitations

Despite the strengths of this study, there are a few limitations that prompt the need for further research. First, the study sample was limited to the perspectives and experiences of adults who communicated verbally. Including the perspectives of autistic adults in middle adulthood was important as a first step to generate further knowledge about PA participation with this group. Given that there are many autistic people who communicate in ways other than verbal speech (Rigles, 2021), future research needs to include participants who communicate in different ways to understand their PA experiences. Including their perspectives is vital as there is a significant dearth of research in this area, yet much is needed. Second, the study sample predominantly comprised late-diagnosed females. Despite recruitment efforts to speak to autistic adults across all identities, our sample had a noticeable gap in participants who identified as male and diverse identities. While it is critical to include the perspectives of autistic females as their voices have been excluded from PA research (Jachyra et al., 2025; Pearson et al., 2022) future research needs to prioritize gender diverse samples in an effort to support the PA needs and interests of all autistic people. Third, the study sample included the perspectives and experiences of younger middle-aged autistic adults. Despite sampling efforts, there was a lack of participants who were in their later years of adulthood. Future research with this group and research employing a lifespan perspective is important to understand how to best enable autistic people to thrive throughout their lives.

## Conclusion

This study examined the PA participation of autistic adults in middle adulthood and the processes that enhanced, shaped and curtailed the PA participation of autistic adults. With a paucity of research to date, the study findings provide a more comprehensive understanding of autistic adults’ PA participation with new insights regarding neuro-normative assumptions and the importance of trust to support PA. Further depth about the role and impacts of sensory sensitivities is also provided. Findings of the study call on the need to co-produce *neurodivergent-informed PA* to support the diverse needs, interests and abilities of autistic people. The use of TIA to support the PA of autistic people is discussed as a potential tool to create safe places and spaces for autistic adults to engage in PA.

## References

[bibr1-13623613251360862] aan het Rot M. Collins K. A. Fitterling H. L . (2009). Physical exercise and depression. Mount Sinai Journal of Medicine: A Journal of Translational and Personalized Medicine, 76(2), 204–214. 10.1002/msj.20094 19306383

[bibr2-13623613251360862] Alhowikan A. (2016). Benefits of physical activity for autism spectrum disorders: A systematic review. Saudi Journal of Sports Medicine, 16(3), 163. 10.4103/1319-6308.187558

[bibr3-13623613251360862] Arkesteyn A. Cornelissen V. Steyaert J. Vancampfort D. Van Damme T. (2025). Barriers and facilitators of physical activity participation in adolescents with autism. Children’s Health Care, 54, 127–170. 10.1080/02739615.2023.2228693

[bibr4-13623613251360862] Bauman A. E. Reis R. S. Sallis J. F. Wells J. C. Loos R. J. F. Martin B. W. (2012). Correlates of physical activity: Why are some people physically active and others not? The Lancet, 380(9838), 258–271. 10.1016/s0140-6736(12)60735-1 22818938

[bibr5-13623613251360862] Benson S. Bender A. M. Wickenheiser H. Naylor A. Clarke M. Samuels C. H. Werthner P. (2018). Differences in sleep patterns, sleepiness, and physical activity levels between young adults with autism spectrum disorder and typically developing controls. Developmental Neurorehabilitation, 22(3), 164–173. 10.1080/17518423.2018.1501777 30067414

[bibr6-13623613251360862] Blagrave A. J. Colombo-Dougovito A. M. Healy S. (2021). ‘Just invite us’: Autistic adults’ recommendations for developing more accessible physical activity opportunities. Autism in Adulthood, 3(2), 179–186. 10.1089/aut.2020.0055 36601469 PMC8992896

[bibr7-13623613251360862] Braun V. Clarke V. (2019). Reflecting on reflexive thematic analysis. Qualitative Research in Sport, Exercise and Health, 11(4), 589–597. 10.1080/2159676x.2019.1628806

[bibr8-13623613251360862] Braun V. Clarke V. (2020). One size fits all? What counts as quality practice in (reflexive) thematic analysis? Qualitative Research in Psychology, 18(3), 328–352. 10.1080/14780887.2020.1769238

[bibr9-13623613251360862] Campbell S. Greenwood M. Prior S. Shearer T. Walkem K. Young S. Bywaters D. Walker K. (2020). Purposive sampling: Complex or simple? Research case examples. Journal of Research in Nursing, 25(8), 652–661. 10.1177/1744987120927206 34394687 PMC7932468

[bibr10-13623613251360862] Campos C. Duck M. McQuillan R. Brazill L. Malik S. Hartman L. McPherson A. C. Gibson B. E. Jachyra P. (2019). Exploring the role of physiotherapists in the care of children with autism spectrum disorder. Physical & Occupational Therapy in Pediatrics, 39(6), 614–628. 30957621 10.1080/01942638.2019.1585405

[bibr11-13623613251360862] Cappadocia M. C. Weiss J. A. Pepler D. (2011). Bullying experiences among children and youth with autism spectrum disorders. Journal of Autism and Developmental Disorders, 42(2), 266–277. 10.1007/s10803-011-1241-x 21499672

[bibr12-13623613251360862] Colombo-Dougovito A. M. Blagrave A. J. Healy S. (2020). A grounded theory of adoption and maintenance of physical activity among autistic adults. Autism, 25(3), 627–641. 10.1177/1362361320932444 32579029

[bibr13-13623613251360862] DeJesus B. M. Oliveira R. C. de Carvalho F. O. de Jesus Mari J. Arida R. M. Teixeira-Machado L. (2020). Dance promotes positive benefits for negative symptoms in autism spectrum disorder (ASD): A systematic review. Complementary Therapies in Medicine, 49, 102299. 10.1016/j.ctim.2020.102299 32147081

[bibr14-13623613251360862] De Lyon A. T. Neville R. D. Armour K. M . (2016). The role of fitness professionals in public health: A review of the literature. Quest, 69(3), 313–330. 10.1080/00336297.2016.1224193

[bibr15-13623613251360862] Duquette M.-M. Carbonneau H. Roult R. Crevier L. (2016). Sport and physical activity: Facilitating interventions with young people living with an autism spectrum disorder. Physical Activity Review, 4, 40–49.

[bibr16-13623613251360862] Elliott R. O. Dobbin A. R. Rose G. D. Soper H. V. (1994). Vigorous, aerobic exercise versus General Motor Training Activities: Effects on maladaptive and stereotypic behaviors of adults with both autism and mental retardation. Journal of Autism and Developmental Disorders, 24(5), 565–576. 10.1007/bf02172138 7814306

[bibr17-13623613251360862] Finch T. L. Mackintosh J. Petrou A. McConachie H. Le Couteur A. Garland D. Parr J. R. (2022). ‘We couldn’t think in the box if we tried. We can’t even find the damn box’: A qualitative study of the lived experiences of autistic adults and relatives of autistic adults. PLOS ONE, 17(3), Article e0264932. 10.1371/journal.pone.0264932 PMC8920184 35286347

[bibr18-13623613251360862] Fortuna R. J. Robinson L. Smith T. H. Meccarello J. Bullen B. Nobis K. Davidson P. W. (2015). Health conditions and functional status in adults with autism: A cross-sectional evaluation. Journal of General Internal Medicine, 31(1), 77–84. 10.1007/s11606-015-3509-x 26361965 PMC4700008

[bibr19-13623613251360862] García-Villamisar D. A. Dattilo J. (2010). Effects of a leisure programme on quality of life and stress of individuals with ASD. Journal of Intellectual Disability Research, 54(7), 611–619. 10.1111/j.1365-2788.2010.01289.x 20500784

[bibr20-13623613251360862] Gibbs V. Pellicano E. (2023). ‘Maybe we just seem like easy targets’: A qualitative analysis of autistic adults’ experiences of interpersonal violence. Autism, 27(7), 2021–2034. 10.1177/13623613221150375 36691297

[bibr21-13623613251360862] Gregor S. Bruni N. Grkinic P. Schwartz L. McDonald A. Thille P. Gabison S. Gibson B. E. Jachyra P. (2018). Parents’ perspectives of physical activity participation among Canadian adolescents with Autism Spectrum Disorder. Research in Autism Spectrum Disorders, 48, 53–62.

[bibr22-13623613251360862] Hamm J. Yun J. (2017). Influence of physical activity on the health-related quality of life of young adults with and without autism spectrum disorder. Disability and Rehabilitation, 41(7), 763–769. 10.1080/09638288.2017.1408708 29182033

[bibr23-13623613251360862] Healy S. Brewer B. Laxton P. Powers B. Daly J. McGuire J. Patterson F. (2021). Brief report: Perceived barriers to physical activity among a national sample of autistic adults. Journal of Autism and Developmental Disorders, 52(10), 4583–4591. 10.1007/s10803-021-05319-8 34623582

[bibr24-13623613251360862] Hillier A. Buckingham A. Schena D. (2020). Physical activity among adults with autism: Participation, attitudes, and barriers. Perceptual and Motor Skills, 127(5), 874–890. 10.1177/0031512520927560 32443953

[bibr25-13623613251360862] Hillier A. Murphy D. Ferrara C. (2011). A pilot study: Short-term reduction in salivary cortisol following low level physical exercise and relaxation among adolescents and young adults on the autism spectrum. Stress and Health, 27(5), 395–402. 10.1002/smi.1391

[bibr26-13623613251360862] Jachyra P. Anagnostou E. Knibbe T. J. Petta C. Cosgrove S. Chen L. Capano L. Moltisanti L. McPherson A. C. (2019). ‘Girls don’t have big tummies’: The experiences of weight-related discussions for children with autism spectrum disorders. Autism: The International Journal of Research and Practice, 23(5), 1096–1105. 30244587 10.1177/1362361318793020

[bibr27-13623613251360862] Jachyra P. McLeod J. Green J. Van Damme T. (2025). Autistic females’ experiences with physical activity [Manuscript Submitted for Publication].

[bibr28-13623613251360862] Jachyra P. McLeod J. Rosenbaum S. (2024). Interview and arts-based approaches for research with autistic adults. In McMahon J. McGannon K. R. (Eds.), Trauma-informed Research in sport, exercise, and health (1st ed.; Vol. 1; pp. 176–194). Routledge.

[bibr29-13623613251360862] Jachyra P. Renwick R. Gladstone B. Anagnostou E. Gibson B. E. (2021). Physical activity participation among adolescents with autism spectrum disorder. Autism: The International Journal of Research and Practice, 25(3), 613–626. 32921151 10.1177/1362361320949344

[bibr30-13623613251360862] Kim B. Lee D. Min A. Paik S. Frey G. Bellini S. Han K. Shih P. C. (2020). PuzzleWalk: A theory-driven iterative design inquiry of a mobile game for promoting physical activity in adults with autism spectrum disorder. PLOS ONE, 15(9), Article e0237966. 10.1371/journal.pone.0237966 PMC7482920 32911501

[bibr31-13623613251360862] Lachman M. E. (2004). Development in midlife. Annual Review of Psychology, 55(1), 305–331. 10.1146/annurev.psych.55.090902.141521 14744218

[bibr32-13623613251360862] Lachman M. E. (Ed.). (2001). Handbook of midlife development. Wiley.

[bibr33-13623613251360862] Lai M.-C. Kassee C. Besney R. Bonato S. Hull L. Mandy W. Szatmari P. Ameis S. H. (2019). Prevalence of co-occurring mental health diagnoses in the autism population: A systematic review and meta-analysis. The Lancet Psychiatry, 6(10), 819–829. 10.1016/S2215-0366(19)30289-5 31447415

[bibr34-13623613251360862] Lochbaum M. Crews D. (2003). Viability of cardiorespiratory and muscular strength programs for the adolescent with autism. Complementary Health Practice Review, 8(3), 225–233. 10.1177/1076167503252917

[bibr35-13623613251360862] Lunsky Y. Lai M. Balogh R. Chung H. Durbin A. Jachyra P. Tint A. Weiss J. Lin E. (2022). Premature mortality in a population-based cohort of autistic adults in Canada. Autism Research, 15(8), 1550–1559. 10.1002/aur.2741 35633154

[bibr36-13623613251360862] MacLennan K. Woolley C. Andsensory E. Heasman B. Starns J. George B. Manning C. (2023). ‘It is a big spider web of things’: Sensory experiences of autistic adults in public spaces. Autism in Adulthood, 5(4), 411–422. 10.1089/aut.2022.0024 38116051 PMC10726197

[bibr37-13623613251360862] Mason D. McConachie H. Garland D. Petrou A. Rodgers J. Parr J. R. (2018). Predictors of quality of life for autistic adults. Autism Research, 11(8), 1138–1147. 10.1002/aur.1965 29734506 PMC6220831

[bibr38-13623613251360862] Nagata J. M. Vittinghoff E. Gabriel K. P. Rana J. S. Garber A. K. Moran A. E. Reis J. P. Lewis C. E. Sidney S. Bibbins-Domingo K. (2022). Physical activity from young adulthood to middle age and premature cardiovascular disease events: A 30-year population-based cohort study. International Journal of Behavioral Nutrition and Physical Activity, 19, 123. 10.1186/s12966-022-01357-2 36127703 PMC9487136

[bibr39-13623613251360862] Nichols C. Block M. E. Bishop J. C. McIntire B. (2018). Physical activity in young adults with autism spectrum disorder: Parental perceptions of barriers and facilitators. Autism, 23(6), 1398–1407. 10.1177/1362361318810221 30486668

[bibr40-13623613251360862] Otter. (2023). Voice meeting notes & real–time transcription. Otter.ai – Voice Meeting Notes & Real-time Transcription. https://otter.ai/

[bibr41-13623613251360862] Parsons K. Payne S. Holt N. Wallace J. (2024). A qualitative study of physical activity drivers in autistic individuals using COM-B. Autistic and non-autistic perspectives. Research in Autism Spectrum Disorders, 111, 102331. 10.1016/j.rasd.2024.102331

[bibr42-13623613251360862] Paudel S. Owen A. J. Smith B. J. (2021). Socio-ecological influences of leisure-time physical activity among Nepalese adults: A qualitative study. BMC Public Health, 21(1), 1443. 10.1186/s12889-021-11484-3 34294069 PMC8296660

[bibr43-13623613251360862] Pearson A. Rees J. Forster S. (2022). ‘This was just how this friendship worked’: Experiences of interpersonal victimization among autistic adults. Autism in Adulthood, 4(2), 141–150. 10.1089/aut.2021.0035 36605970 PMC9645672

[bibr44-13623613251360862] Pellicano E. Stears M. (2011). Bridging autism, science and society: Moving toward an ethically informed approach to autism research. Autism Research, 4(4), 271–282. 10.1002/aur.201 21567986

[bibr45-13623613251360862] Peterson J. L. Earl R. K. Fox E. A. Ma R. Haidar G. Pepper M. Berliner L. Wallace A. S. Bernier R. A. (2019). Trauma and autism spectrum disorder: Review, proposed treatment adaptations and future directions. Journal of Child & Adolescent Trauma, 12(4), 529–547. 10.1007/s40653-019-00253-5 31819782 PMC6901292

[bibr46-13623613251360862] Pinckard K. Baskin K. K. Stanford K. I. (2019). Effects of exercise to improve cardiovascular health. Frontiers in Cardiovascular Medicine, 6, Article 69. 10.3389/fcvm.2019.00069 PMC6557987 31214598

[bibr47-13623613251360862] Piven J. Rabins P. (2011). Autism spectrum disorders in older adults: Toward defining a research agenda. Journal of the American Geriatrics Society, 59(11), 2151–2155. 10.1111/j.1532-5415.2011.03632.x 22091837

[bibr48-13623613251360862] Reeve D. (2012). Psycho-emotional disablism: The missing link? In Watson N. Roulstone A. Thomas C. (Eds.), Routledge handbook of disability studies (pp. 78–92). Routledge. http://donnareeve.co.uk/wp-content/uploads/2014/03/ReeveChapter2012b.pdf

[bibr49-13623613251360862] Rigles B. (2021). Trajectories of adverse childhood experiences among children with autism. Research in Autism Spectrum Disorders, 89, 101876. 10.1016/j.rasd.2021.101876

[bibr50-13623613251360862] Sallis J. F. Cervero R. B. Ascher W. Henderson K. A. Kraft M. K. Kerr J. (2006). An ecological approach to creating active living communities. Annual Review of Public Health, 27(1), 297–322. 10.1146/annurev.publhealth.27.021405.102100 16533119

[bibr51-13623613251360862] SAMHSA. (2014). SAMHSA’s concept of trauma and guidance for a trauma-informed approach. https://library.samhsa.gov/sites/default/files/sma14-4884.pdf

[bibr52-13623613251360862] Schwartz-Shea P. Yanow D. (2013). Interpretive research design: Concepts and processes. Routledge.

[bibr53-13623613251360862] Shahane V. Kilyk A. Srinivasan S. M. (2023). Effects of physical activity and exercise-based interventions in young adults with autism spectrum disorder: A systematic review. Autism, 28(2), 276–300. 10.1177/13623613231169058 37128159

[bibr54-13623613251360862] Shahrasfenghar A. Arabameri E. Daneshfar A. Ghasemi A. Kashi A. (2019). The effect of aerobic exercise on motor skills and body composition of children with autism. Journal of Health and Care, 20(4), 332–341. 10.29252/jhc.20.4.332

[bibr55-13623613251360862] Smith B. Roderick M. Hockin-Boyers H. Monforte J. Williams T. L. Jachyra P. Dodd-Reynolds C. Phoenix C. (2025). 25 years of qualitative research in sport and exercise Psychology: How did it Go? and what now?. Psychology of Sport and Exercise, 81, 102958. 10.1016/j.psychsport.2025.102958 40712908

[bibr56-13623613251360862] Smith B. Sparkes A. C. (2016). Interviews: Qualitative interviewing in the sport and exercise sciences. In Smith B. Sparkes A. C. (Eds.), Routledge handbook of qualitative research in sport and exercise (pp. 125–145). Routledge. 10.4324/9781315762012-19

[bibr57-13623613251360862] Sorensen C. Zarrett N. (2014). Benefits of physical activity for adolescents with autism spectrum disorders: A comprehensive review. Review Journal of Autism and Developmental Disorders, 1(4), 344–353. 10.1007/s40489-014-0027-4

[bibr58-13623613251360862] Sowa M. Meulenbroek R. (2012). Effects of physical exercise on autism spectrum disorders: A meta-analysis. Research in Autism Spectrum Disorders, 6(1), 46–57. 10.1016/j.rasd.2011.09.001

[bibr59-13623613251360862] Tse C. Y. Lee H. P. Chan K. S. Edgar V. B. Wilkinson-Smith A. Lai W. H. (2019). Examining the impact of physical activity on sleep quality and executive functions in children with autism spectrum disorder: A randomized controlled trial. Autism, 23(7), 1699–1710. 10.1177/1362361318823910 30663324

[bibr60-13623613251360862] Waldron D. A. Stokes J. Coyle C. E. Kramer J. Dugan E. (2023). Aging on the autism spectrum: Physical activity in individuals receiving state services in the United States. Journal of Autism and Developmental Disorders, 53, 3943–3957. 10.1007/s10803-022-05676-y 35933645

[bibr61-13623613251360862] Weir E. Allison C. Warrier V. Baron-Cohen S. (2020). Increased prevalence of non-communicable physical health conditions among autistic adults. Autism, 25(3), 681–694. 10.1177/1362361320953652 32907337 PMC7610707

[bibr62-13623613251360862] Woods R. Waltz M. (2019). The strength of autistic expertise and its implications for autism knowledge production: A response to Damian Milton. https://shura.shu.ac.uk/24752/1/Woods_StrengthofAutistic%28AM%29.pdf

[bibr63-13623613251360862] Wright Stein S. Alexander R. Mann J. Schneider C. Zhang S. Gibson B. E. Gabison S. Jachyra P. Mosleh D . (2022). Understanding disability in healthcare: Exploring the perceptions of parents of young people with autism spectrum disorder. Disability and Rehabilitation, 44(19), 5623–5630. 34232798 10.1080/09638288.2021.1948114

